# Keeping synapses in shape: degradation pathways in the healthy and aging brain

**DOI:** 10.1042/NS20210063

**Published:** 2022-06-15

**Authors:** Marijn Kuijpers

**Affiliations:** 1 Leibniz-Forschungsinstitut für Molekulare Pharmakologie (FMP), Robert-Rössle-Straße 10, 13125 Berlin, Germany; 2 Department of Molecular Neurobiology, Donders Institute for Brain, Cognition and Behaviour and Faculty of Science, Radboud University, Nijmegen, The Netherlands

**Keywords:** aging, autophagy, endolysosome, presynapse, proteostasis, ubiquitin proteasome system

## Abstract

Synapses maintain their molecular composition, plasticity and function through the concerted action of protein synthesis and removal. The complex and polarized neuronal architecture poses specific challenges to the logistics of protein and organelle turnover since protein synthesis and degradation mainly happen in the cell soma. In addition, post-mitotic neurons accumulate damage over a lifetime, challenging neuronal degradative pathways and making them particularly susceptible to the effects of aging. This review will summarize the current knowledge on neuronal protein turnover mechanisms with a particular focus on the presynapse, including the proteasome, autophagy and the endolysosomal route and their roles in regulating presynaptic proteostasis and function. In addition, the author will discuss how physiological brain aging, which entails a progressive decline in cognitive functions, affects synapses and the degradative machinery.

## Introduction

**Figure 1:**
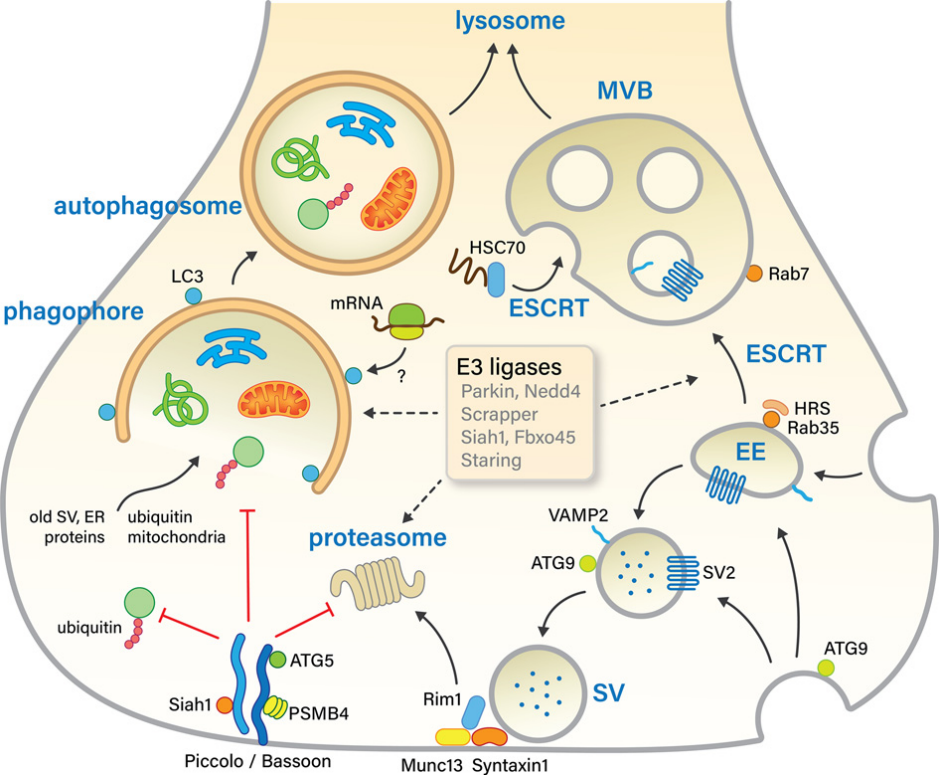
Degradative pathways and suggested presynaptic cargos Synaptic proteins and organelles, such as SVs and mitochondria, can be locally removed via alternative degradative routes. The proteasome removes predominantly cytosolic proteins, including active zone proteins RIM1 and Munc13. ESCRT-dependent endolysosomal sorting is thought to be required for the degradation of a subset of membrane proteins, for instance, SV proteins VAMP2 and SV2. Both proteins and organelles can be sequestered by a double-membrane structure called a phagophore, which then closes to form an autophagosome that eventually fuses with somatic lysosomes for degradation. Ubiquitination is a common signal for degradation in all of these major protein degradation pathways. Therefore, ubiquitin-ligases (E3) and deubiquitinating enzymes could play important roles in maintaining a healthy presynapse. The six E3 ligases indicated here either have known, mammalian, presynaptic targets (Nedd4, Scrapper, Fbxo45, Staring) or are locally regulated by presynaptic proteins (Siah1, Parkin); EE, early endosome; SV, synaptic vesicle; MVB, multivesicular body.

Protein homeostasis, or proteostasis, refers to all protein synthesis and degradation processes that preserve the essential balance of cellular protein levels. Synapses are especially dependent on the mechanisms for maintaining proteostasis as they contain an extremely dense network of proteins that needs to undergo constant remodeling to govern neurotransmission and synapse reshaping during learning and memory processes [1,2]. Most synapses are located far away from the soma, the main compartment for protein synthesis and degradation, creating extra challenges to the logistics of proteostasis pathways. In addition, because of their post-mitotic and long-lived nature, neurons and their synapses are at increased risk for age-related damage accumulation. During aging, cells encounter stressful situations, caused for instance by a buildup of unfolded proteins or an accumulation of reactive oxygen species (ROS) causing lipid peroxidation and protein oxidation [3]. At the synapse, high metabolic burdens and calcium insults during neuronal activity might even accelerate protein damages and turnover [4,5]. The burden on mature and aging synapses increases even more, as aging is associated with a progressive decline in the efficiency of degradation systems, such as the proteasome, endolysosomal and autophagy pathway. This review will first summarize current knowledge on the proteostasis processes that act to remove dysfunctional synaptic components (Figure 1) and how, for instance, these pathways are locally regulated to maintain proper functioning of the presynapse. Second, this review will address how physiological brain aging affects the synapse and its degradative capacity.

## Ubiquitin–proteasome system (UPS)

A central feature of all major degradative pathways is the ability to selectivity target substrates by site-specific mono- and poly-ubiquitination. Ubiquitin is a 76-amino acid protein that covalently attaches to substrate proteins. This conjugation relies on the sequential activities of three classes of enzymes: E1 (ubiquitin-activating), E2 (ubiquitin-conjugating) and E3 (ubiquitin ligases). Ubiquitination is initiated by E1, an enzyme requiring ATP for the activation of ubiquitin. Ubiquitin is then handed over to an E2 to form an ubiquitin∼E2 conjugate via a thioester exchange reaction. The (>600 known) E3 ligases mediate the final step of ubiquitin transfer and provide the specificity for substrate recognition by interacting with both E2 and the target protein [6]. Ubiquitin can create different types of poly-ubiquitin chains via its lysine residues and each type of chain has the potential to act as a distinct intracellular signal. For instance, K48-linked chains are a prime signal for the ubiquitin–proteasome system (UPS) whereas K63-linked chains play a more prominent role in lysosomal degradation [7]. Deubiquitination enzymes (DUBs) serve as an important regulatory layer and contribute to the replenishment of ubiquitin pools.

The highly conserved UPS is responsible for the degradation of the majority of short-lived, cytosolic proteins. The proteasome consists of a barrel-shaped 20S catalytic core particle and 19S regulatory particles that select and unfold target proteins labeled with ubiquitin chains. During presynaptic assembly, a local decrease in UPS activity is proposed to aid in the formation of a stable cluster of synaptic vesicles (SVs) [8–10]. Research over the years also demonstrate critical functions for the UPS in mature synapses, with UPS involvement in the regulation of basal neurotransmission and synaptic plasticity [2,11,12]. Synaptic plasticity includes long-term potentiation (LTP) and long-term depression (LTD) that are thought to form the cellular basis of learning and memory, and their dysfunction may underlie memory loss and other cognitive disorders [13,14]. Incubation of hippocampal slices with proteasome inhibitors enhances early phase LTP induction [15] while impairing late phase LTP maintenance [11,16]. Bilateral infusion of lactacystin, a specific proteasome inhibitor, in the hippocampal CA1 area of rats is shown to impair memory formation and memory extinction [17,18]. The mechanisms that underlie proteasome-dependent plasticity have not been fully elucidated but probably depend on changes in the postsynaptic compartment [17,19]. In addition, proteasome inhibition is implicated in presynaptic neurotransmitter release. At the *Drosophila* neuromuscular synapse, acute pharmacological inhibition of the proteasome in the presynaptic neuron results in stabilization of active zone protein Unc-13/Munc13 and increased synaptic transmission due to a more efficient priming and release of SVs [20]. In cultured rat hippocampal neurons, proteasome inhibition causes an increase in the size of the recycling pool, although in this study no effect on SV release was observed [21]. Proteasome 20S complexes are mainly present in the cytosol, but a recent finding indicates that they can associate with the plasma membrane of neurons, where they are involved in the activity-dependent degradation of intracellular proteins, producing extracellular peptides that can modulate neuronal activity [22]. It is currently unclear how these surface-exposed proteasomes target to the plasma membrane and are embedded into the lipid bilayer.

Apart from changes in neurotransmission, proteasome dysregulation in mature synapses also causes structural presynaptic abnormalities. Inactivation of PI31, an adaptor for microtubule-based transport of proteasomes, results in the accumulation of ubiquitinated protein aggregates in mouse and fly axon terminals and is accompanied by distal axon swellings and presynapse loss [23,24], while leaving the postsynapse mostly unperturbed [23]. Whereas in these studies the identity of the accumulated proteins was not determined, other works have indicated that the levels of some presynaptic proteins, such as synaptophysin, liprin-alpha, RIM, Bassoon and above-mentioned Munc13 are controlled via UPS-mediated degradation [25–28]. At the mature mammalian presynapse, ubiquitin E3 ligases have been identified to induce proteasomal degradation of presynaptic proteins (Figure 1). The E3 enzyme Staring, for instance, ubiquitinates syntaxin 1, a member of the SNARE complex and an essential component of the SV fusion machinery [29]. E3 ligases SCRAPPER and Fbxo45 ubiquitinate active zone proteins RIM1 and Munc13, thereby contributing to the UPS-dependent regulation of SV release. Accordingly, loss of these E3 ligases results in a facilitation of neurotransmission [30,31]. In addition, the presynaptic active zone (AZ) proteins Bassoon and Piccolo are known to bind and inhibit the E3 ligase Siah1 [32]. Bassoon has also been found to interact with PSMB4, a structural subunit of the 20S core proteasome, thereby interfering with compartment-specific ubiquitination and proteasome biogenesis [33]. Accordingly, loss of Bassoon and Piccolo increases ubiquitination and degradation of SV proteins, eventually resulting in SV loss and synapse elimination. Thus, these AZ proteins seem to stabilize the AZ by inhibiting degradation. Interestingly, core AZ components show age-induced increases in *Drosophila* brains [34], but whether mammalian neurons show similar trends is unknown. Regarding the role of synaptic deubiquitylation very little is known. At the postsynapse, USP46 is identified as the DUB for AMPARs, stabilizing cell-surface expression levels [35]. Our knowledge regarding presynaptic ubiquitin regulation, including the identity, roles and substrates of DUBs is still very limited and thus warrants further investigation.

While the UPS locally and preferentially targets short-lived soluble proteins, the endolysosomal and autophagy pathways are responsible for the elimination of membrane proteins, long-lived proteins, insoluble protein aggregates and dysfunctional organelles [36]. In neuronal axons and synapses, both the endolysosomal and autophagy pathways are retrograde trafficking routes that deliver proteins to degradative lysosomes that are primarily present in neuronal somata [37].

## The endolysosomal pathway

Transmembrane proteins and associated factors are typically degraded by the endolysosomal system and access this pathway by endocytosis. Pre-existing early endosomes then fuse with these endocytosed vesicles, permitting proteins to recycle back to the cell surface (regulated by small GTPases Rab4 and Rab11) or directing cargo to late endosomes or late endosomal multivesicular body (MVB) and finally to lysosomes for degradation (regulated by Rab7) [38]. During their transport towards the soma, axonal endosomes gradually maturate and acidify which may also control their degradative capacity [37,39]. In the pathway promoting degradation, Endosomal Sorting Complexes Required for Transport (ESCRT) is a core protein machinery, important for the recognition of ubiquitinated membrane cargo and the formation, trafficking, and fusion of MVBs. The ESCRT machinery is composed of multiprotein complexes known as ESCRT-0, I, II and III. The ESCRT-0 protein HRS initiates the process by binding to ubiquitinated cargos and tethering them to the surface of early endosomes by binding phosphatidylinositol 3-phosphate (PI[3]P). On endosomes, PI[3]P is generated by the Vps34 phosphatidylinositol 3-kinase complex II (Vps34/Vps15/Beclin1) that assembles at early endosomes via the association of Vps15 with Rab5 [40]. Recruitment of Rab5 effectors, such as Rabaptin-5 and Rabex-5, can regulate Rab5 activity and the formation of a local endosomal PI3P pool [41]. The sequential recruitment and activation of the other ESCRT complexes promotes inward vesicle budding and membrane sealing by the Vps4 ATPase [42]. Studies on ESCRT-dependent presynaptic protein degradation are scarce but evidence from *Drosophila* and cultured rat hippocampal neurons suggests that endosomal sorting is required for the degradation of a subset of ubiquitinated SV proteins (e.g. VAMP2 and SV2), a process involving active Rab35 dependent HRS recruitment to SV pools [43,44]. In agreement with this, mutations in CHMP2B, an ESCRT-III subunit involved in frontotemporal dementia, results in an increase in presynaptic endosomes and a selective retention of SV-associated proteins in aged mice [45]. In contrast, at the *Drosophila* photoreceptor axon terminals, sorting of SV proteins was found to be independent from Rab7 whereas degradative sorting of plasma membrane proteins was Rab7-dependent, indicating the involvement of multiple degradative sorting pathways [46]. Cytosolic proteins can also enter the endolysosomal system by “endosomal microautophagy”, a pathway dependent on ESCRTs, but not the core autophagy machinery (see below). This process relies on the cytosolic chaperone Hsc70 for substrate targeting and the subsequent invagination-mediated uptake by late endosomes/MVBs. In *Drosophila* neurons, endosomal microautophagy mediates the turnover of synaptic proteins harboring a KFERQ recognition motif, including for instance Unc-13/Munc13 and EndophilinA, thereby rejuvenating the protein pool and facilitating synaptic transmission [47]. Given the fact that in the presynapse most organelles and markers for the early stages of the endolysosomal route are present, including MVBs, Rabs and ESCRT components [48–50], this route might play an underappreciated but important role in synaptic proteostasis and function and therefore represent an interesting area for future research.

## Autophagy at the presynapse

Although often used synonymously, there are at least three distinct types of autophagy; microautophagy, chaperone-mediated autophagy (CMA) and macroautophagy. Microautophagy (similar to endosomal microautophagy) is defined by the direct uptake of cargo by the lysosome via lysosomal membrane invagination [51]. In CMA, selective cytosolic substrate proteins bind the chaperone Hsc70 and are thought to be translocated across the lysosomal membrane via the receptor lysosome-associated membrane protein type 2A (LAMP2A) [52]. The most prevalent form of autophagy, however, is macroautophagy (hereafter called autophagy) that utilizes double membrane vesicles, called autophagosomes, that deliver their content to the lysosome. Under baseline conditions, neuronal autophagosomes are mostly generated in the axon tip and retrogradely transported to the cell soma for fusion with degradative lysosomes [53]. Autophagosome formation requires a “core machinery” that starts with initiation factor ULK1 kinase complex, which translocates to the endoplasmic reticulum (ER) membrane (or another membrane donor) to activate the VPS34/ATG14/Beclin1 complex, that in turn triggers the production of PI[3]P. The subsequent recruitment of the PI[3]P effector WIPI2B allows for further recruitment of autophagy-specific ubiquitin-like conjugation systems, including E1-like ATG7 and the E3-like ATG12-ATG5:ATG16L ligase complex, that lipidate LC3 and LC3-related proteins. The lipidated form of LC3 stably inserts itself into the growing inner and outer autophagosome membrane to promote membrane elongation, substrate recruitment and finally autophagosome closure [54]. Autophagy can be either a non-selective bulk process, or a selective process that utilizes so-called autophagy receptors that can recognize substrates and simultaneously interact with lipidated LC3, targeting specific substrates to the inner membrane of the growing autophagosome. The specific action of these receptors is regulated by protein modifications, such as ubiquitination, phosphorylation and oligomerization [55]. Autophagosomes are then transported to the soma in a dynein-dependent manner, a process that is linked to their maturation and gradual acidification, for example by fusion with late endosomes or lysosomes to form autolysosomes [56]. Interestingly, the canonical machinery involved in synaptic transmission can also act to locally regulate presynaptic autophagy. Presynaptic endocytic proteins Endophilin and Synaptojanin, for instance, promote autophagosome formation by aiding the lipidation of LC3 and autophagosome closure while the endocytic adaptor complex AP2 facilitates retrograde axonal transport of mature autophagosomes (also referred to as amphisomes) to promote TrkB/BDNF signaling [57]. Conversely, the AZ protein Bassoon inhibits autophagy by binding to autophagy protein ATG5 [58–61].

Autophagosome biogenesis and transport in axons have been studied in detail [62], and emerging studies now tackle the question what substrates are targeted by neuronal autophagy and how this process is involved in maintaining synapse function. At the post-synapse, recent studies show that dendritic autophagy is required for synaptic LTD and the removal of postsynaptic AMPA receptor subunits and scaffold proteins [63,64]. The main sites of autophagosome biogenesis are at distal axons where autophagosomes are proposed to contain cargoes such as damaged SV proteins [65,66] but also mitochondria [67,68], ER [69] and whole SVs [67,70]. Similar to proteins, organelles have finite lifetimes and accumulate damages over time that can, if not taken care of, lead to organelle dysfunction. In addition, during endo-exocytotic cycles, SVs are gradually contaminated with proteins that normally reside on the plasma membrane. According to one hypothesis the accumulation of a certain number of these proteins prevents aged SVs from fusion with the PM. Hence, such aged SVs need to be degraded [71]. In mouse dopaminergic neurons, induction of autophagy by treatment with the mTOR inhibitor rapamycin results in an ATG7-dependent reduction in the number of SVs and a consecutive decrease in evoked neurotransmitter release [72], suggesting that SVs as a whole can be targeted to the autophagy pathway. The synaptic vesicle-associated GTPase Rab26 is postulated to connect SV recycling and autophagy by binding to autophagy protein ATG16L and directing SVs as a whole to pre-autophagosomal structures [70,73]. *Rab26* null mutant fruit flies suffer from defective stimulus-dependent neurotransmission, although this did not appear to be accompanied by changes in autophagosomal and SV markers, suggesting a possible autophagy-independent function of Rab26 [74]. Recent work indicates a role for ATG9, the only known transmembrane protein involved in the core autophagy pathway, by linking the activity-dependent SV cycle with autophagosome formation. ATG9 exhibits lipid scramblase activity and, together with the lipid transport protein ATG2, mediates the flow of lipids from a donor membrane to the growing autophagosome membrane [75]. ATG9 is enriched at presynaptic terminals where it plays a role in local autophagosome biogenesis [76]. Recent work in *Caenorhabditis elegans* and mouse primary neurons shows that ATG9-containing vesicles undergo exo-endocytosis at presynaptic sites in an activity-dependent manner, via shared components of the machinery for SV recycling. Mutants that disrupt synaptic endocytic traffic, including a Parkinson’s disease (PD) associated mutation in synaptojanin1/unc-26, result in presynaptic ATG9 accumulation and accelerate the age-dependent decay of *C. elegans* synapses and locomotor behavior [77]. How ATG9 cycling aides in autophagosome biogenesis and what autophagy substrates underlie the reported age-dependent changes remains to be explored.

Modulation of presynaptic neurotransmission by autophagy is not only proposed to occur via removal of SVs and their associated proteins. Knockout of the core autophagy protein ATG5 selectively in excitatory forebrain neurons results in increased excitatory neurotransmission at CA3-CA1 synapses in acute mouse hippocampal slices and in primary hippocampal cultures, an effect independent from SV number or SV protein levels [69]. Instead, the gain in neurotransmission results from elevated calcium release from endoplasmic reticulum (ER) tubules, which accumulates at presynaptic sites as a consequence of abolished autophagy. Another independent study also failed to detect major alterations in the levels of SV proteins in brains lacking ATG5 specifically in either excitatory or inhibitory neurons. Instead, they identified autophagy-dependent degradation of synaptic subunits of protein kinase A (PKA), a key regulatory enzyme involved in the phosphorylation of the synaptic proteome [78]. In contrast, a recent study using proteomic analysis of isolated autophagic vesicles from wild-type mouse brain tissue suggested synaptic proteins as basal autophagy cargoes [67]. This may reflect differences between the analysis of cargos under wild-type conditions or cargo accumulation in autophagy inhibited condition, a situation in which other mechanisms might be able to take over degradation of certain components. The same study revealed mitochondrial fragments as a major cargo within neuronal autophagosomes. Due to their role in sequestering cytosolic calcium or ATP production, presynaptic mitochondria can regulate presynaptic release, plasticity and increase synapse stability [79,80], suggesting that autophagy-mediated removal of axonal mitochondria modulates presynaptic processes. Indeed, in aged rodents and fruit flies, mitochondria lose their functionality possible as a consequence of malfunctional autophagy [81,82] (and see further below). Future studies will be needed to determine how synaptic cargo is recognized and what autophagy receptors and protein modifications might aid substrate recognition.

To allow neuronal synapses to modify their proteome during processes like plasticity, regulated protein degradation, but also protein synthesis must be implemented (for a nice overview on synaptic protein synthesis and delivery see [83]). Traditionally believed to be a purely somatic event, synthesis of proteins from messenger RNA (mRNA) can occur locally at the distal presynapse [84]. Interestingly, a recent study shows that the autophagy protein LC3B is an RNA-binding protein that can trigger rapid mRNA degradation upon autophagic activation in HEK293T cells, thereby mediating cross-talk between autophagy and local protein synthesis [85]. It would be interesting to see if LC3-dependent RNA degradation plays a role in regulating the local synaptic proteome.

## Common signals and regulators of degradative pathways

Degradative pathways can respond to a multitude of stressors, including protein damage and nutrient starvation. These signaling pathways can regulate degradation pathways in different ways. For example, inhibition of the mechanistic target of rapamycin (mTOR) signaling is responsible for autophagy induction during nutrient starvation and neuronal activity [86–88] but is also involved in proteasomal inhibition [89]. Interestingly, mTOR is present at synapses. Here, its inactivation decreases the strength of evoked excitatory synaptic transmission via both post- and presynaptic mechanisms, including enhanced spontaneous SV vesicle fusion [90]. Another signal that can influence both UPS and autophagy is proteostatic imbalance in the ER (known as ER stress). ER stress can be a result of abnormalities in calcium homeostasis, oxidative stress and other conditions leading to protein folding defects, processes postulated to play an important role in the rate of aging. To restore balance, ER stress activates many different pathways, including autophagy and the unfolded protein response (UPR), a stress response mediated by ER transmembrane stress sensors (PERK, ATF6 and IRE1α) that induces transcriptional and translational programs to lower protein production and promote degradation. ER stress and the UPR are commonly associated with neurodegenerative diseases [91], and UPR manipulations can restore cognition and synapse function in aged mice [92,93]. Recent studies suggest that UPR can be locally initiated by activity in the distal dendrite and at postsynapses, thereby inducing dendritic growth [94], but the exact contributions of this broad process to synapse maintenance are presently unknown.

Although the above discussed degradation pathways use different molecular machineries, some of the key players are shared between them. A common denominator of all is the selection of substrates by ubiquitination. Ubiquitin ligases as well as DUBs can be shared between the degradation pathways. The E3 ubiquitin ligase PRKN/Parkin for instance, can catalyze a range of ubiquitination events, including mono-ubiquitination, K48- and K63-linked poly-ubiquitination. These events can mark substrates for the proteasome [95] but also play an important role in autophagic degradation of mitochondria [96] and, as more recently suggested, presynaptic vesicle autophagy [97]. Another E3 ligase, named Nedd4, induces rapid mGlu7 degradation via both proteasomal and lysosomal pathways [98]. Similarly, ubiquitin receptors can convey selectivity but are sometimes involved in multiple pathways, for instance, the autophagy receptor p62 brings ubiquitinated proteins to the autophagosome via LC3 binding but is also involved in proteasomal degradation [99]. As indicated above, the large AZ protein Bassoon can modulate the presynaptic proteome in several ways, by negatively regulating autophagy [60], proteasome activity [33] and protein ubiquitination [32]. Components of the degradative pathways are themselves often also substrates, proteasomes for example, can be degraded by bulk autophagy [100]. In addition, the MVB and autophagy pathways merge at the late endosome/lysosome [101], making both pathways sensitive to a block in lysosomal degradation by, for instance, proton pump or phosphoinositide 3-kinase (PI3K) inhibitors. Thus, when targeting specific protein degradation pathways or proteins, effects on downstream machineries or redundancy of degradative pathways need to be taken into consideration.

## Global synapse changes during normal aging

Even in the absence of neurodegenerative diseases, the functional capabilities of our brain, such as memory and processing speed, progressively decline with age. Synapses form the requisite connections of the neuronal networks that underlie cognition and therefore represent an important target for the effects of aging. Human synapse formation starts prenatally and lasts until about 2 years after birth, after which half of all synapses are subsequently “pruned” during the following two decades [102]. After this developmental period, a proportion of synaptic connections will be stably maintained over life spans of months to years [103]. Other connections, however, are unstable with turnover rates in the range of a few days to weeks, likely depending on the brain region [104–106]. Alterations in synaptic turnover are correlated with the ability to learn and to retain information, indicating the importance for neuronal circuit plasticity [107,108]. A growing body of evidence supports the idea that disruptions in these synaptic connections drive brain changes and cognitive decline that occur during normal aging [109]. Decreases in spine density have been reported in aging rodents [92,110–112] (however also see [113,114]), non-human primates [115], and humans [116]. *In vivo* two-photon imaging of cortical spines and boutons in old mice (>20 months) revealed changes in basal synapse dynamics but not number [116,117] with for instance a reduction in learning-induced new spine formation [111]. The hippocampus, an area essential for learning and episodic memory, is particularly sensitive to aging [118] and a reduction in SV numbers is observed in aged hippocampal mossy fiber (MF)-CA3, and CA3-CA1 synapses [119]. These structural synaptic changes observed during aging also lead to functional and behavioral deteriorations. Electrophysiological studies in the context of aging consistently show changes in synaptic transmission, excitability and plasticity. In general, aging is often associated with a decreased synaptic transmission and a reduced ability to increase synaptic strength through LTP [120,121]. Not all synapses, however, are equally affected by aging. For instance, aged rodents display defective presynaptic LTP at MF-CA3 synapses [119] and reduced excitability in CA1 [122] but hyperactivation in CA3 pyramidal neurons [123]. A recent study combining electron microscopy and behavioral experiments, set out to link hippocampal CA1 and CA3 synaptic architecture with cognitive ability during aging. They demonstrated that synapse architecture did not change in old rats that were performing as well in learning-tasks as their young counterparts. In contrast, old but learning-impaired animals exhibited structural synaptic changes only in CA3 synapses. Notably, synapses within hippocampal region CA3 showed an increased number of perforated synapses, a synapse characterized by gaps in post- and presynaptic densities, and a different distribution of synaptic weight as measured by AMPAR expression [124]. Thus, although in some brain regions specific neuronal loss is observed during aging processes [125], it is clearly the more subtle changes in plastic synaptic connections that drive cognitive decline during normal aging. Other studies set out to address what molecular changes occur that could underlie these age-induced changes in synaptic connections. Studies have shown age-related decline of synaptic proteins such as synaptophysin [126] and NMDA receptor subunits [127]. Recently, multiple large datasets have become available that include age-related neuron and synapse changes in mice, monkeys and humans on multiple levels including the transcriptome [128–133], proteome [92,134–136], lipidome [137], epigenome [138] and metabolome [139]. These studies now provide an important basis for identifying signatures for cell changes during aging; however, we still lack detailed understanding on what underlies these changes and how they can be directly related to synaptic connectivity changes and cognitive decline during aging.

In agreement with previous studies in *C. elegans* [140,141], a recent study employing *in vivo* metabolic labeling and proteomic profiling of mouse brains, correlated normal aging of wild-type mice with decreases in global protein recycling [134]. Why global proteostasis declines during normal aging processes is not well understood but includes decreases in the efficiency and cooperation of protein quality control systems. The next paragraphs will focus on how physiological aging affects the major presynaptic protein degradation routes.

## Age-related changes in presynaptic degradation pathways

During normal brain aging, malfunctioning of autophagy processes are described in many organisms such as *D. melanogaster*, rodents, and humans. This includes changes in mTOR activity, gene expression, down-regulation of autophagy proteins (including ATG5, ATG7 and Beclin), and decreases in the amount of autophagosomes or autophagic flux [142–146]. A number of studies have shown that autophagy contributes to memory formation [63,147,148] and emerging studies in mice demonstrate a link between age-induced impairment of hippocampus-dependent memories and the down-regulation of certain autophagy factors, highlighting the need for functional neuronal autophagy in learning and memory. Importantly, genetic manipulations restoring autophagic function are sufficient to rescue age-related memory decline [147]. Similarly, the autophagy inducer spermidine protects from age-related synaptic alterations at murine hippocampal MF-CA3 synapses and counteracts age-dependent memory impairment in mice and fruit flies [82,119,149]. Moreover, elevated dietary spermidine intake is associated with a lower risk for cognitive impairment and decline in humans [82]. How autophagy is differentially modulated at aging presynapses remains unclear. Age-induced increases in the levels of the active zone scaffolding protein Bassoon might inhibit autophagy and, thereby, reduce release probability and synaptic transmission. Studies performed in primary mouse dorsal root ganglion neurons have revealed an age-dependent decrease in autophagosome biogenesis, caused by a local loss of the PI[3]P effector WIPI2B. This leads to the stalling of autophagosomes and failure to recruit LC3 at axon terminals. Strikingly, overexpression of WIPI2 was sufficient to return axonal autophagosome biogenesis to levels observed in younger neurons [150]. Whether hippocampal synapses experience similar age-associated autophagy impairments remains to be determined.

The relatively newly discovered chaperone-mediated autophagy (CMA) also decreases with age, predominantly due to changes in lysosomal lipid composition leading to reduced stability of LAMP2A [151]. Recent work on rodent models with neuron-specific LAMP2A loss demonstrated changes reminiscent of brain aging, including accumulation of oxidized and ubiquitinated proteins, synapse loss and memory impairments [52,152]. Although presynaptic accumulation of α-synuclein is observed after CMA loss [153], there has been little research on CMA at synapses and its role in modulating synaptic content and function needs to be determined.

Defects in the neuronal endosome-lysosome and ESCRT pathway, including pH increases and enlargement of early endosomes, are one of the earliest observed changes in neurodegenerative diseases such as Alzheimer’s disease (AD) and PD [154]. However, few data are available on changes in this pathway during normal brain aging. Recent findings identified the BAG3-HSP70 complex as a mediator of endolysosomal degradation in rat cortical neurons, exerting its effects by disinhibiting Rab35 activity [155]. Interestingly, BAG3 expression is decreased in AD brain pathology [156] but is increased during normal aging [157]. Whereas ESCRT proteins control the pathway towards lysosomal degradation, another protein complex called Retromer (i.e. comprising the proteins Vps35, Vps26 and Vps29) mediates the recycling of membrane proteins to the Golgi complex or back to the cell surface. The association of the core Retromer complex with different sorting nexin (SNX) proteins provides Retromer diversity in terms of cargo recognition and trafficking pathways [158]. Retromer cargos include for instance postsynaptic neurotransmitter receptors [159,160] and the dopamine transporter DAT, a presynaptic protein required for dopamine clearance following release [161]. Although little is known about how aging influences Retromer efficiency, an age-dependent decrease of most Retromer components is observed in the mouse cortex [162]. When Retromer-dependent processes are disrupted, many protein cargoes are misdirected to the degradative pathway, leading to improper recycling of for instance lysosomal cathepsins and neurodegeneration [163]. Recent studies in *Drosophila* suggest that presynaptic Retromer regulates SV recycling [164,165], although Vps35 depletion in primary hippocampal mouse neurons did not alter synaptic vesicle release or retrieval [166] .

In the aging brain, the UPS is proposed to be modified in different ways, including alterations in the ubiquitin-conjugating and DUB enzyme landscape [167–170] and the expression or transport of proteasome subunits [171,172]. The accumulation of protein inclusions due to proteostasis failure can also clog and inactivate the proteasome [173]. This proteostasis feedback during aging makes it difficult to dissect primary causes, such as changes in UPS expression, from secondary consequences due to protein overload. In synaptosomal fractions from aged monkey brains, levels of ubiquitin-conjugating enzyme UBE2N increase with aging while UPS activity declines, potentially leading to an accumulation of ubiquitinated and misfolded proteins in neuronal terminals [170]. Similarly, proteasome activity is decreased during aging in *Drosophila*, and enhancement of neuronal proteasome function prolongs lifespan and delays age-related decline in memory and learning [174], although the background mechanisms are so far unknown. Little is known about age-related UPS changes and their impact on cognition in humans. A recent proteomic study on enriched synaptic fractions from human brain samples connected the levels of proteasome subunits to cognitive performance [135]. Here, proteasome subunit levels were decreased in subjects that, despite AD pathology, are cognitively unimpaired but elevated in aged individuals with low pathology but with impaired cognition. In conclusion, whereas most studies indicate age-related decline in proteasome function, the relationship between cognitive aging and impairment of the UPR are far from clear.

## Conclusions and open questions

In recent years, we have gained fundamental insight into the degradative mechanisms involved in synaptic protein turnover and their relevance for neurotransmission. Both basal and stress-evoked pathways can damage synapse integrity and further research will be vital to uncover how synaptic components such as aged SVs are recognized and tagged for degradation. In addition, how do different regulators and signals such as neuronal activity, mTOR and ER-stress impinge on local degradative pathways in the synapse? How is the synaptic availability and activity of ubiquitin ligases and DUBs regulated, and how do all these different degradative pathways cooperate to sustain synapse integrity?

Much of what we know about aging comes from rodents, mostly from transgenic mouse model of age-related neurodegenerative diseases such as AD and PD. However, emerging studies show different, and often opposite, effects on the brain during neuropathology as compared with normal aging, indicating that neurodegenerative diseases are not a mere acceleration of aging. Whereas protein recycling in the brain decreases in aged, but otherwise healthy, mice, increased protein turnover is associated with aging in, for instance, mouse models of Alzheimer’s disease [134]. Here, differential effects on degradative pathways might account for the discrepancy. For instance during pathology an unusual high amount of misfolded proteins can clog the proteasome and other degradative pathways. It is also important to recognize that individual synapses show high degree of diversity arising from molecular and morphological differences [175]. We know that region-specific vulnerability underlies pathological brain aging, with for instance the hippocampus primarily affected in Alzheimer’s disease, the substantia nigra in Parkinson’s disease and the ventral spinal cord in amyotrophic lateral sclerosis [176]. During physiological aging, changes in synaptic architecture, composition, and function also vary depending on brain region and synapse type [110,124]. In addition, not all brain areas experience the same age-related changes in degradative pathways [177], indicating that physiological brain aging is as diverse as pathological brain aging. Therefore, we have to take into account that population-averaging measurements might not be able to detect all changes that happen with age. What is underlying this differential vulnerability of neuronal and synaptic populations is unclear but likely depends on intrinsic differences between synapses, such as their activity, their turnover rates or dependence on, and availability of, certain proteolytic systems. Recent studies also suggest cell extrinsic factors to play a role in age-dependent synapse decline, including systemic factors such as microbial metabolites (reviewed in [178]). How environmental factors shape protein homeostasis in humans is still an open debate but non-pharmacological methods such as caloric restriction and physical exercise increase neuronal plasticity and resistance of neurons to age-related decline in humans, possibly via common pathways such as mTOR and autophagy [179]. Finding the molecular pathways involved in synapse aging will be essential to design future interventions to halt or even reverse brain aging.

## Data Availability

No data were generated for this review.

## Abbreviations

AD: Alzheimer’s disease
CMA: chaperone-mediated autophagy
mTOR: mechanistic target of rapamycin
PD: Parkinson’s disease
PI3K: phosphoinositide 3-kinase
PKA: protein kinase A
ROS: reactive oxygen species
UPR: unfolded protein response
UPS: ubiquitin–proteasome system

## References

[1] Cajigas I.J., Will T. and Schuman E.M. (2010) Protein homeostasis and synaptic plasticity. EMBO J. 29, 2746–2752 10.1038/emboj.2010.173 20717144 PMC2924649

[2] Hegde A.N. (2017) Proteolysis, synaptic plasticity and memory. Neurobiol. Learn. Mem. 138, 98–110 10.1016/j.nlm.2016.09.003 27614141 PMC5336522

[3] Sonninen T.M., Goldsteins G., Laham-Karam N., Koistinaho J. and Lehtonen S. (2020) Proteostasis disturbances and inflammation in neurodegenerative diseases. Cells 9, 10.3390/cells9102183 32998318 PMC7601929

[4] Pulido C. and Ryan T.A. (2021) Synaptic vesicle pools are a major hidden resting metabolic burden of nerve terminals. Sci. Adv. 7, eabi9027 10.1126/sciadv.abi9027 34860552 PMC8641928

[5] Heo S., Diering G.H., Na C.H., Nirujogi R.S., Bachman J.L., Pandey A. et al. (2018) Identification of long-lived synaptic proteins by proteomic analysis of synaptosome protein turnover. Proc. Natl. Acad. Sci. U.S.A. 115, E3827–E3836 10.1073/pnas.1720956115 29610302 PMC5910858

[6] Zheng N. and Shabek N. (2017) Ubiquitin ligases: structure, function, and regulation. Annu. Rev. Biochem. 86, 129–157 10.1146/annurev-biochem-060815-014922 28375744

[7] Kocaturk N.M. and Gozuacik D. (2018) Crosstalk between mammalian autophagy and the ubiquitin-proteasome system. Front. Cell Dev. Biol. 6, 10.3389/fcell.2018.00128 30333975 PMC6175981

[8] Hamilton A.M., Oh W.C., Vega-Ramirez H., Stein I.S., Hell J.W., Patrick G.N. et al. (2012) Activity-dependent growth of new dendritic spines is regulated by the proteasome. Neuron 74, 1023–1030 10.1016/j.neuron.2012.04.031 22726833 PMC3500563

[9] Pinto M.J., Alves P.L., Martins L., Pedro J.R., Ryu H.R., Jeon N.L. et al. (2016) The proteasome controls presynaptic differentiation through modulation of an on-site pool of polyubiquitinated conjugates. J. Cell Biol. 212, 789–801 10.1083/jcb.201509039 27022091 PMC4810304

[10] Valnegri P., Huang J., Yamada T., Yang Y., Mejia L.A., Cho H.Y. et al. (2017) RNF8/UBC13 ubiquitin signaling suppresses synapse formation in the mammalian brain. Nat. Commun. 8, 1271 10.1038/s41467-017-01333-6 29097665 PMC5668370

[11] Fonseca R., Vabulas R.M., Hartl F.U., Bonhoeffer T. and Nagerl U.V. (2006) A balance of protein synthesis and proteasome-dependent degradation determines the maintenance of LTP. Neuron 52, 239–245 10.1016/j.neuron.2006.08.015 17046687

[12] Turker F., Cook E.K. and Margolis S.S. (2021) The proteasome and its role in the nervous system. Cell Chem Biol. 28, 903–917 10.1016/j.chembiol.2021.04.003 33905676 PMC8286317

[13] Radulescu C.I., Cerar V., Haslehurst P., Kopanitsa M. and Barnes S.J. (2021) The aging mouse brain: cognition, connectivity and calcium. Cell Calcium 94, 102358 10.1016/j.ceca.2021.102358 33517250

[14] Bliss T.V. and Collingridge G.L. (1993) A synaptic model of memory: long-term potentiation in the hippocampus. Nature 361, 31–39 10.1038/361031a0 8421494

[15] Dong C., Upadhya S.C., Ding L., Smith T.K. and Hegde A.N. (2008) Proteasome inhibition enhances the induction and impairs the maintenance of late-phase long-term potentiation. Learn. Mem. 15, 335–347 10.1101/lm.984508 18441292 PMC2364605

[16] Karpova A., Mikhaylova M., Thomas U., Knopfel T. and Behnisch T. (2006) Involvement of protein synthesis and degradation in long-term potentiation of Schaffer collateral CA1 synapses. J. Neurosci. 26, 4949–4955 10.1523/JNEUROSCI.4573-05.2006 16672670 PMC6674165

[17] Lee S.H., Choi J.H., Lee N., Lee H.R., Kim J.I., Yu N.K. et al. (2008) Synaptic protein degradation underlies destabilization of retrieved fear memory. Science 319, 1253–1256 10.1126/science.1150541 18258863

[18] Lopez-Salon M., Alonso M., Vianna M.R.M., Viola H., Souza T.M.E., Izquierdo I. et al. (2001) The ubiquitin-proteasome cascade is required for mammalian long-term memory formation. Eur. J. Neurosci. 14, 1820–1826 10.1046/j.0953-816x.2001.01806.x 11860477

[19] Ehlers M.D. (2003) Activity level controls postsynaptic composition and signaling via the ubiquitin-proteasome system. Nat. Neurosci. 6, 231–242 10.1038/nn1013 12577062

[20] Speese S.D., Trotta N., Rodesch C.K., Aravamudan B. and Broadie K. (2003) The ubiquitin proteasome system acutely regulates presynaptic protein turnover and synaptic efficacy. Curr. Biol. 13, 899–910 10.1016/S0960-9822(03)00338-5 12781128

[21] Willeumier K., Pulst S.M. and Schweizer F.E. (2006) Proteasome inhibition triggers activity-dependent increase in the size of the recycling vesicle pool in cultured hippocampal neurons. J. Neurosci. 26, 11333–11341 10.1523/JNEUROSCI.1684-06.2006 17079661 PMC2665188

[22] Ramachandran K.V., Fu J.M., Schaffer T.B., Na C.H., Delannoy M. and Margolis S.S. (2018) Activity-dependent degradation of the nascentome by the neuronal membrane proteasome. Mol. Cell. 71, 169e6–177e6 10.1016/j.molcel.2018.06.013 29979964 PMC6070390

[23] Liu K., Jones S., Minis A., Rodriguez J., Molina H. and Steller H. (2019) PI31 is an adaptor protein for proteasome transport in axons and required for synaptic development. Dev. Cell. 50, 509e10–524e10 10.1016/j.devcel.2019.06.009 31327739 PMC6702053

[24] Minis A., Rodriguez J.A., Levin A., Liu K., Govek E.E., Hatten M.E. et al. (2019) The proteasome regulator PI31 is required for protein homeostasis, synapse maintenance, and neuronal survival in mice. Proc. Natl. Acad. Sci. U. S. A 116, 24639–24650 10.1073/pnas.1911921116 31754024 PMC6900516

[25] Jiang X.P., Litkowski P.E., Taylor A.A., Lin Y., Snider B.J. and Moulder K.L. (2010) A role for the ubiquitin-proteasome system in activity-dependent presynaptic silencing. J. Neurosci. 30, 1798–1809 10.1523/JNEUROSCI.4965-09.2010 20130189 PMC2824895

[26] Lazarevic V., Schone C., Heine M., Gundelfinger E.D. and Fejtova A. (2011) Extensive remodeling of the presynaptic cytomatrix upon homeostatic adaptation to network activity silencing. J. Neurosci. 31, 10189–10200 10.1523/JNEUROSCI.2088-11.2011 21752995 PMC6623065

[27] Vitry S., Ausseil J., Hocquemiller M., Bigou S., Dos Santos Coura R. and Heard J.M. (2009) Enhanced degradation of synaptophysin by the proteasome in mucopolysaccharidosis type IIIB. Mol. Cell. Neurosci. 41, 8–18 10.1016/j.mcn.2009.01.001 19386237

[28] Hakim V., Cohen L.D., Zuchman R., Ziv T. and Ziv N.E. (2016) The effects of proteasomal inhibition on synaptic proteostasis. EMBO J. 35, 2238–2262 10.15252/embj.201593594 27613546 PMC5069550

[29] Chin L.S., Vavalle J.P. and Li L. (2002) Staring, a novel E3 ubiquitin-protein ligase that targets syntaxin 1for degradation. J. Biol. Chem. 277, 35071–35079 10.1074/jbc.M203300200 12121982

[30] Tada H., Okano H.J., Takagi H., Shibata S., Yao I., Matsumoto M. et al. (2010) Fbxo45, a novel ubiquitin ligase, regulates synaptic activity. J. Biol. Chem. 285, 3840–3849 10.1074/jbc.M109.046284 19996097 PMC2823526

[31] Yao I., Takagi H., Ageta H., Kahyo T., Sato S., Hatanaka K. et al. (2007) SCRAPPER-dependent ubiquitination of active zone protein RIM1 regulates synaptic vesicle release. Cell 130, 943–957 10.1016/j.cell.2007.06.052 17803915 PMC3049808

[32] Waites C.L., Leal-Ortiz S.A., Okerlund N., Dalke H., Fejtova A., Altrock W.D. et al. (2013) Bassoon and Piccolo maintain synapse integrity by regulating protein ubiquitination and degradation. EMBO J. 32, 954–969 10.1038/emboj.2013.27 23403927 PMC3616282

[33] Montenegro-Venegas C., Fienko S., Anni D., Pina-Fernández E., Frischknecht R. and Fejtova A. (2021) Bassoon inhibits proteasome activity via interaction with PSMB4. Cell. Mol. Life Sci. 78, 1545–1563 10.1007/s00018-020-03590-z 32651614 PMC7904567

[34] Gupta V.K., Pech U., Bhukel A., Fulterer A., Ender A., Mauermann S.F. et al. (2016) Spermidine suppresses age-associated memory impairment by preventing adverse increase of presynaptic active zone size and release. PLoS Biol. 14, e1002563 10.1371/journal.pbio.1002563 27684064 PMC5042543

[35] Huo Y., Khatri N., Hou Q., Gilbert J., Wang G. and Man H.Y. (2015) The deubiquitinating enzyme USP46 regulates AMPA receptor ubiquitination and trafficking. J. Neurochem. 134, 1067–1080 10.1111/jnc.13194 26077708 PMC4668950

[36] Klionsky D.J. (2007) Autophagy: from phenomenology to molecular understanding in less than a decade. Nat. Rev. Mol. Cell Bio. 8, 931–937 10.1038/nrm2245 17712358

[37] Kuijpers M., Azarnia Tehran D., Haucke V. and Soykan T. (2021) The axonal endolysosomal and autophagic systems. J. Neurochem. 158, 589–602 10.1111/jnc.15287 33372296

[38] Huotari J. and Helenius A. (2011) Endosome maturation. Embo J. 30, 3481–3500 21878991 10.1038/emboj.2011.286 PMC3181477

[39] Overly C.C. and Hollenbeck P.J. (1996) Dynamic organization of endocytic pathways in axons of cultured sympathetic neurons. J. Neurosci. 16, 6056–6064 10.1523/JNEUROSCI.16-19-06056.1996 8815888 PMC6579168

[40] Wallroth A. and Haucke V. (2018) Phosphoinositide conversion in endocytosis and the endolysosomal system. J. Biol. Chem. 293, 1526–1535 10.1074/jbc.R117.000629 29282290 PMC5798284

[41] Borchers A.C., Langemeyer L. and Ungermann C. (2021) Who's in control? Principles of Rab GTPase activation in endolysosomal membrane trafficking and beyond J. Cell Biol. 220, 10.1083/jcb.202105120 34383013 PMC8366711

[42] Vietri M., Radulovic M. and Stenmark H. (2020) The many functions of ESCRTs. Nat. Rev. Mol. Cell Biol. 21, 25–42 10.1038/s41580-019-0177-4 31705132

[43] Sheehan P., Zhu M., Beskow A., Vollmer C. and Waites C.L. (2016) Activity-dependent degradation of synaptic vesicle proteins requires Rab35 and the ESCRT pathway. J. Neurosci. 36, 8668–8686 10.1523/JNEUROSCI.0725-16.2016 27535913 PMC4987437

[44] Uytterhoeven V., Kuenen S., Kasprowicz J., Miskiewicz K. and Verstreken P. (2011) Loss of skywalker reveals synaptic endosomes as sorting stations for synaptic vesicle proteins. Cell 145, 117–132 10.1016/j.cell.2011.02.039 21458671

[45] Clayton E.L., Bonnycastle K., Isaacs A.M., Cousin M.A. and Schorge S. (2022) A novel synaptopathy-defective synaptic vesicle protein trafficking in the mutant CHMP2B mouse model of frontotemporal dementia. J. Neurochem. 160, 412–425 10.1111/jnc.15551 34855215

[46] Jin E.J., Kiral F.R., Ozel M.N., Burchardt L.S., Osterland M., Epstein D. et al. (2018) Live observation of two parallel membrane degradation pathways at axon terminals. Curr. Biol. 28, 1027.e4–1038.e4 10.1016/j.cub.2018.02.032 29551411 PMC5944365

[47] Uytterhoeven V., Lauwers E., Maes I., Miskiewicz K., Melo M.N., Swerts J. et al. (2015) Hsc70-Hsc74 deforms membranes to promote synaptic protein turnover by endosomal microautophagy. Neuron 88, 735–748 10.1016/j.neuron.2015.10.012 26590345

[48] Birdsall V., Zhu M., Kirwan K., Imoto Y., Watanabe S. and Waites C.L. (2021) Axonal transport of Hrs is activity-dependent and rate limiting for synaptic vesicle protein degradation. bioRxiv 2020.04.16.044818 10.26508/lsa.202000745 PMC9152131 35636965

[49] Ivanova D. and Cousin M.A. (2022) Synaptic vesicle recycling and the endolysosomal system: a reappraisal of form and function. Front. Synaptic Neurosci. 14, 826098 10.3389/fnsyn.2022.826098 35280702 PMC8916035

[50] Turegano-Lopez M., Santuy A., DeFelipe J. and Merchan-Perez A. (2020) Size, shape, and distribution of multivesicular bodies in the juvenile rat somatosensory cortex: a 3D electron microscopy study. Cereb. Cortex 30, 1887–1901 10.1093/cercor/bhz211 31665237 PMC7132939

[51] Schuck S. (2020) Microautophagy - distinct molecular mechanisms handle cargoes of many sizes. J. Cell Sci. 133, jcs246322 10.1242/jcs.246322 32907930

[52] Bourdenx M., Gavathiotis E. and Cuervo A.M. (2021) Chaperone-mediated autophagy: a gatekeeper of neuronal proteostasis. Autophagy 17, 2040–2042 10.1080/15548627.2021.1935007 34110247 PMC8386735

[53] Cheng X.T., Zhou B., Lin M.Y., Cai Q. and Sheng Z.H. (2015) Axonal autophagosomes use the ride-on service for retrograde transport toward the soma. Autophagy 11, 1434–1436 10.1080/15548627.2015.1062203 26102591 PMC4590659

[54] Melia T.J., Lystad A.H. and Simonsen A. (2020) Autophagosome biogenesis: From membrane growth to closure. J. Cell Biol. 219, e202002085 10.1083/jcb.202002085 32357219 PMC7265318

[55] Gubas A. and Dikic I. (2022) A guide to the regulation of selective autophagy receptors. FEBS J. 289, 75–89 10.1111/febs.15824 33730405

[56] Cason S.E., Carman P.J., van Duyne C., Goldsmith J., Dominguez R. and Holzbaur E.L.F. (2021) Sequential dynein effectors regulate axonal autophagosome motility in a maturation-dependent pathway. J. Cell Biol. 220, e202010179 10.1083/jcb.202010179 34014261 PMC8142281

[57] Kononenko N.L., Claßen G.A., Kuijpers M., Puchkov D., Maritzen T., Tempes A. et al. (2017) Retrograde transport of TrkB-containing autophagosomes via the adaptor AP-2 mediates neuronal complexity and prevents neurodegeneration. Nat. Commun. 8, 14819 10.1038/ncomms14819 28387218 PMC5385568

[58] Soukup S.F., Kuenen S., Vanhauwaert R., Manetsberger J., Hernández-Díaz S., Swerts J. et al. (2016) A LRRK2-dependent EndophilinA phosphoswitch is critical for macroautophagy at presynaptic terminals. Neuron 92, 829–844 10.1016/j.neuron.2016.09.037 27720484

[59] Hernandez-Diaz S., Ghimire S., Sanchez-Mirasierra I., Montecinos-Oliva C., Swerts J., Kuenen S. et al. (2022) Endophilin-B regulates autophagy during synapse development and neurodegeneration. Neurobiol. Dis. 163, 105595 10.1016/j.nbd.2021.105595 34933093

[60] Okerlund N.D., Schneider K., Leal-Ortiz S., Montenegro-Venegas C., Kim S.A., Garner L.C. et al. (2017) Bassoon controls presynaptic autophagy through Atg5. Neuron 93, 897–+ 10.1016/j.neuron.2017.01.026 28231469

[61] Vanhauwaert R., Kuenen S., Masius R., Bademosi A., Manetsberger J., Schoovaerts N. et al. (2017) The SAC1 domain in synaptojanin is required for autophagosome maturation at presynaptic terminals. EMBO J. 36, 1392–1411 10.15252/embj.201695773 28331029 PMC5430236

[62] Stavoe A.K.H. and Holzbaur E.L.F. (2019) Axonal autophagy: Mini-review for autophagy in the CNS. Neurosci. Lett. 697, 17–23 10.1016/j.neulet.2018.03.025 29548988 PMC6136980

[63] Compans B., Camus C., Kallergi E., Sposini S., Martineau M., Butler C. et al. (2021) NMDAR-dependent long-term depression is associated with increased short term plasticity through autophagy mediated loss of PSD-95. Nat. Commun. 12, 2849 10.1038/s41467-021-23133-9 33990590 PMC8121912

[64] Kallergi E., Daskalaki A.D., Kolaxi A., Camus C., Ioannou E., Mercaldo V. et al. (2022) Dendritic autophagy degrades postsynaptic proteins and is required for long-term synaptic depression in mice. Nat. Commun. 13, 680 10.1038/s41467-022-28301-z 35115539 PMC8814153

[65] Hill S.E., Kauffman K.J., Krout M., Richmond J.E., Melia T.J. and Colon-Ramos D.A. (2019) Maturation and clearance of autophagosomes in neurons depends on a specific cysteine protease isoform, ATG-4.2. Dev. Cell. 49, 251–266, e8 10.1016/j.devcel.2019.02.013 30880001 PMC6482087

[66] Hoffmann S., Orlando M., Andrzejak E., Bruns C., Trimbuch T., Rosenmund C. et al. (2019) Light-Activated ROS Production Induces Synaptic Autophagy. J. Neurosci. 39, 2163–2183 10.1523/JNEUROSCI.1317-18.2019 30655355 PMC6433757

[67] Goldsmith J., Ordureau A., Harper J.W. and Holzbaur E.L.F. (2022) Brain-derived autophagosome profiling reveals the engulfment of nucleoid-enriched mitochondrial fragments by basal autophagy in neurons. Neuron 110, 967–976 10.1016/j.neuron.2021.12.029 35051374 PMC8930448

[68] Maday S. and Holzbaur E.L. (2012) Autophagosome assembly and cargo capture in the distal axon. Autophagy 8, 858–860 10.4161/auto.20055 22617438 PMC3378425

[69] Kuijpers M., Kochlamazashvili G., Stumpf A., Puchkov D., Swaminathan A., Lucht M.T. et al. (2022) Neuronal autophagy regulates presynaptic neurotransmission by controlling the axonal endoplasmic reticulum. Neuron 110, 734 10.1016/j.neuron.2022.01.029 35176244 PMC8860378

[70] Binotti B., Pavlos N.J., Riedel D., Wenzel D., Vorbrüggen G., Schalk A.M. et al. (2015) The GTPase Rab26 links synaptic vesicles to the autophagy pathway. Elife 4, e05597 10.7554/eLife.05597 25643395 PMC4337689

[71] Truckenbrodt S., Viplav A., Jähne S., Vogts A., Denker A., Wildhagen H. et al. (2018) Newly produced synaptic vesicle proteins are preferentially used in synaptic transmission. EMBO J. 37, e98044 10.15252/embj.201798044 29950309 PMC6068464

[72] Hernandez D., Torres C.A., Setlik W., Cebrián C., Mosharov E.V., Tang G. et al. (2012) Regulation of presynaptic neurotransmission by macroautophagy. Neuron 74, 277–284 10.1016/j.neuron.2012.02.020 22542182 PMC3578406

[73] Lüningschrör P., Binotti B., Dombert B., Heimann P., Perez-Lara A., Slotta C. et al. (2017) Plekhg5-regulated autophagy of synaptic vesicles reveals a pathogenic mechanism in motoneuron disease. Nat. Commun. 8, 678 10.1038/s41467-017-00689-z 29084947 PMC5662736

[74] Kohrs F.E., Daumann I.M., Pavlovic B., Jin E.J., Kiral F.R., Lin S.C. et al. (2021) Systematic functional analysis of rab GTPases reveals limits of neuronal robustness to environmental challenges in flies. Elife 10, e59594 10.7554/eLife.59594 33666175 PMC8016483

[75] Nakatogawa H. (2020) Mechanisms governing autophagosome biogenesis. Nat. Rev. Mol. Cell Biol. 21, 439–458 10.1038/s41580-020-0241-0 32372019

[76] Stavoe A.K., Hill S.E., Hall D.H. and Colón-Ramos D.A. (2016) KIF1A/UNC-104 transports ATG-9 to regulate neurodevelopment and autophagy at synapses. Dev. Cell. 38, 171–185 10.1016/j.devcel.2016.06.012 27396362 PMC4961624

[77] Yang S., Park D., Manning L., Hill S.E., Cao M., Xuan Z. et al. (2022) Presynaptic autophagy is coupled to the synaptic vesicle cycle via ATG-9. Neuron 110, 824.e10–840.e10 10.1016/j.neuron.2021.12.031 35065714 PMC9017068

[78] Overhoff M., Tellkamp F., Hess S., Tutas J., Tolve M., Faerfers M. et al. (2022) Autophagy regulates neuronal excitability by controlling cAMP/Protein Kinase A signaling. bioRxiv 2022.02.11.480034 10.15252/embj.2022110963 PMC9670194 36217825

[79] Lees R.M., Johnson J.D. and Ashby M.C. (2020) Presynaptic boutons that contain mitochondria are more stable. Front. Synaptic Neuro. 11, 37 10.3389/fnsyn.2019.00037 31998110 PMC6966497

[80] Todorova V. and Blokland A. (2017) Mitochondria and synaptic plasticity in the mature and aging nervous system. Curr. Neuropharmacol. 15, 166–173 10.2174/1570159X14666160414111821 27075203 PMC5327446

[81] Liang Y., Piao C., Beuschel C.B., Toppe D., Kollipara L., Bogdanow B. et al. (2021) eIF5A hypusination, boosted by dietary spermidine, protects from premature brain aging and mitochondrial dysfunction. Cell Rep. 35, 108941 10.1016/j.celrep.2021.108941 33852845

[82] Schroeder S., Hofer S.J., Zimmermann A., Pechlaner R., Dammbrueck C., Pendl T. et al. (2021) Dietary spermidine improves cognitive function. Cell Rep. 35, 108985 10.1016/j.celrep.2021.108985 33852843

[83] Grochowska K.M., Andres-Alonso M., Karpova A. and Kreutz M.R. (2022) The needs of a synapse-How local organelles serve synaptic proteostasis. EMBO J. e110057 35285533 10.15252/embj.2021110057 PMC8982616

[84] Hafner A.S., Donlin-Asp P.G., Leitch B., Herzog E. and Schuman E.M. (2019) Local protein synthesis is a ubiquitous feature of neuronal pre- and postsynaptic compartments. Science 364, 650–+ 10.1126/science.aau3644 31097639

[85] Hwang H.J., Ha H., Lee B.S., Kim B.H., Song H.K. and Kim Y.K. (2022) LC3B is an RNA-binding protein to trigger rapid mRNA degradation during autophagy. Nat. Commun. 13, 1436 10.1038/s41467-022-29139-1 35302060 PMC8931120

[86] Shehata M., Matsumura H., Okubo-Suzuki R., Ohkawa N. and Inokuchi K. (2012) Neuronal stimulation induces autophagy in hippocampal neurons that is involved in AMPA receptor degradation after chemical long-term depression. J. Neurosci. 32, 10413–10422 10.1523/JNEUROSCI.4533-11.2012 22836274 PMC6703735

[87] Raab-Graham K.F., Haddick P.C., Jan Y.N. and Jan L.Y. (2006) Activity- and mTOR-dependent suppression of Kv1.1 channel mRNA translation in dendrites. Science 314, 144–148 10.1126/science.1131693 17023663

[88] Takei N., Furukawa K., Hanyu O., Sone H. and Nawa H. (2014) A possible link between BDNF and mTOR in control of food intake. Front. Psychol. 5, 1093 10.3389/fpsyg.2014.01093 25309497 PMC4174734

[89] Zhao J.H., Zhai B., Gygi S.P. and Goldberg A.L. (2015) mTOR inhibition activates overall protein degradation by the ubiquitin proteasome system as well as by autophagy. Proc. Natl. Acad. Sci. U. S. A. 112, 15790–15797 10.1073/pnas.1521919112 26669439 PMC4703015

[90] McCabe M.P., Cullen E.R., Barrows C.M., Shore A.N., Tooke K.I., Laprade K.A. et al. (2020) Genetic inactivation of mTORC1 or mTORC2 in neurons reveals distinct functions in glutamatergic synaptic transmission. Elife 9, e51440 10.7554/eLife.51440 32125271 PMC7080408

[91] Hetz C. and Saxena S. (2017) ER stress and the unfolded protein response in neurodegeneration. Nat. Rev. Neurol. 13, 477–491 10.1038/nrneurol.2017.99 28731040

[92] Cabral-Miranda F., Tamburini G., Martinez G., Medinas D., Gerakis Y., Miedema T. et al. (2020) Control of mammalian brain aging by the unfolded protein response (UPR). bioRxiv, 10.1101/2020.04.13.039172

[93] Krukowski K., Nolan A., Frias E.S., Boone M., Ureta G., Grue K. et al. (2020) Small molecule cognitive enhancer reverses age-related memory decline in mice. Elife 9, e62048 10.7554/eLife.62048 33258451 PMC7721440

[94] Saito A., Cai L., Matsuhisa K., Ohtake Y., Kaneko M., Kanemoto S. et al. (2018) Neuronal activity-dependent local activation of dendritic unfolded protein response promotes expression of brain-derived neurotrophic factor in cell soma. J. Neurochem. 144, 35–49 10.1111/jnc.14221 28921568 PMC5765399

[95] Yoshii S.R., Kishi C., Ishihara N. and Mizushima N. (2011) Parkin mediates proteasome-dependent protein degradation and rupture of the outer mitochondrial membrane. J. Biol. Chem. 286, 19630–19640 10.1074/jbc.M110.209338 21454557 PMC3103342

[96] Narendra D., Tanaka A., Suen D.F. and Youle R.J. (2008) Parkin is recruited selectively to impaired mitochondria and promotes their autophagy. J. Cell Biol. 183, 795–803 10.1083/jcb.200809125 19029340 PMC2592826

[97] Hoffmann-Conaway S., Brockmann M.M., Schneider K., Annamneedi A., Rahman K.A., Bruns C. et al. (2020) Parkin contributes to synaptic vesicle autophagy in Bassoon-deficient mice. Elife 9, e56590 10.7554/eLife.56590 32364493 PMC7224700

[98] Lee S., Park S., Lee H., Han S., Song J.M., Han D. et al. (2019) Nedd4 E3 ligase and beta-arrestins regulate ubiquitination, trafficking, and stability of the mGlu7 receptor. Elife 8, e44502 10.7554/eLife.44502 31373553 PMC6690720

[99] Liu W.J., Ye L., Huang W.F., Guo L.J., Xu Z.G., Wu H.L. et al. (2016) p62 links the autophagy pathway and the ubiqutin-proteasome system upon ubiquitinated protein degradation. Cell. Mol. Biol. Lett. 21, 29 10.1186/s11658-016-0031-z 28536631 PMC5415757

[100] Kristensen A.R., Schandorff S., Høyer-Hansen M., Nielsen M.O., Jäättelä M., Dengjel J. et al. (2008) Ordered organelle degradation during starvation-induced autophagy. Mol. Cell. Proteomics 7, 2419–2428 10.1074/mcp.M800184-MCP200 18687634

[101] Andres-Alonso M., Kreutz M.R. and Karpova A. (2021) Autophagy and the endolysosomal system in presynaptic function. Cell. Mol. Life Sci. 78, 2621–2639 10.1007/s00018-020-03722-5 33340068 PMC8004491

[102] Petanjek Z., Judaš M., Šimic G., Rasin M.R., Uylings H.B., Rakic P. et al. (2011) Extraordinary neoteny of synaptic spines in the human prefrontal cortex. Proc. Natl. Acad. Sci. U. S. A. 108, 13281–13286 10.1073/pnas.1105108108 21788513 PMC3156171

[103] Grutzendler J., Kasthuri N. and Gan W.B. (2002) Long-term dendritic spine stability in the adult cortex. Nature 420, 812–816 10.1038/nature01276 12490949

[104] Attardo A., Fitzgerald J.E. and Schnitzer M.J. (2015) Impermanence of dendritic spines in live adult CA1 hippocampus. Nature 523, 592–596 10.1038/nature14467 26098371 PMC4648621

[105] Pfeiffer T., Poll S., Bancelin S., Angibaud J., Inavalli V.K., Keppler K. et al. (2018) Chronic 2P-STED imaging reveals high turnover of dendritic spines in the hippocampus in vivo. Elife 7, e34700 10.7554/eLife.34700 29932052 PMC6014725

[106] Quinn D.P., Kolar A., Harris S.A., Wigerius M., Fawcett J.P. and Krueger S.R. (2019) The stability of glutamatergic synapses is independent of activity level, but predicted by synapse size. Front. Cell Neurosci. 13, 291 10.3389/fncel.2019.00291 31316356 PMC6609312

[107] Ash R.T., Fahey P.G., Park J., Zoghbi H.Y. and Smirnakis S.M. (2018) Increased axonal bouton stability during learning in the mouse model of MECP2 duplication syndrome. eNeuro 5, 10.1523/ENEURO.0056-17.2018 PMC6086213 30105297

[108] Holtmaat A. and Caroni P. (2016) Functional and structural underpinnings of neuronal assembly formation in learning. Nat. Neurosci. 19, 1553–1562 10.1038/nn.4418 27749830

[109] Morrison J.H. and Baxter M.G. (2012) The ageing cortical synapse: hallmarks and implications for cognitive decline. Nat. Rev. Neurosci. 13, 240–250 10.1038/nrn3200 22395804 PMC3592200

[110] Cizeron M., Qiu Z., Koniaris B., Gokhale R., Komiyama N.H., Fransen E. et al. (2020) A brainwide atlas of synapses across the mouse life span. Science 369, 270–275 10.1126/science.aba3163 32527927 PMC7115813

[111] Huang L.Y., Zhou H., Chen K., Chen X. and Yang G. (2020) Learning-dependent dendritic spine plasticity is reduced in the aged mouse cortex. Front. Neural Circuit 14, 581435 10.3389/fncir.2020.581435 PMC7726160 33324172

[112] Wallace M., Frankfurt M., Arellanos A., Inagaki T. and Luine V. (2007) Impaired recognition memory and decreased prefrontal cortex spine density in aged female rats. Ann. N. Y. Acad. Sci. 1097, 54–57 10.1196/annals.1379.026 17413010

[113] Davidson A.M., Mejia-Gomez H., Jacobowitz M. and Mostany R. (2020) Dendritic spine density and dynamics of layer 5 pyramidal neurons of the primary motor cortex are elevated with aging. Cereb. Cortex 30, 767–777 10.1093/cercor/bhz124 31298696 PMC7306167

[114] Geinisman Y., Ganeshina O., Yoshida R., Berry R.W., Disterhoft J.F. and Gallagher M. (2004) Aging, spatial learning, and total synapse number in the rat CA1 stratum radiatum. Neurobiol. Aging 25, 407–416 10.1016/j.neurobiolaging.2003.12.001 15123345

[115] Page T.L., Einstein M., Duan H., He Y., Flores T., Rolshud D. et al. (2002) Morphological alterations in neurons forming corticocortical projections in the neocortex of aged Patas monkeys. Neurosci. Lett. 317, 37–41 10.1016/S0304-3940(01)02428-4 11750991

[116] Mostany R., Anstey J.E., Crump K.L., Maco B., Knott G. and Portera-Cailliau C. (2013) Altered synaptic dynamics during normal brain aging. J. Neurosci. 33, 4094–4104 10.1523/JNEUROSCI.4825-12.2013 23447617 PMC6619332

[117] Grillo F.W., Song S., Teles-Grilo Ruivo L.M., Huang L., Gao G., Knott G.W. et al. (2013) Increased axonal bouton dynamics in the aging mouse cortex. Proc. Natl. Acad. Sci. U. S. A. 110, E1514–E1523 10.1073/pnas.1218731110 23542382 PMC3631669

[118] Burke S.N. and Barnes C.A. (2010) Senescent synapses and hippocampal circuit dynamics. Trends Neurosci. 33, 153–161 10.1016/j.tins.2009.12.003 20071039 PMC3076741

[119] Maglione M., Kochlamazashvili G., Eisenberg T., Racz B., Michael E., Toppe D. et al. (2019) Spermidine protects from age-related synaptic alterations at hippocampal mossy fiber-CA3 synapses. Sci. Rep.-Uk 9, 19616 10.1038/s41598-019-56133-3 PMC6927957 31873156

[120] Rex C.S., Kramár E.A., Colgin L.L., Lin B., Gall C.M. and Lynch G. (2005) Long-term potentiation is impaired in middle-aged rats: regional specificity and reversal by adenosine receptor antagonists. J. Neurosci. 25, 5956–5966 10.1523/JNEUROSCI.0880-05.2005 15976084 PMC6724797

[121] Rizzo V., Richman J. and Puthanveettil S.V. (2014) Dissecting mechanisms of brain aging by studying the intrinsic excitability of neurons. Front. Aging Neurosci. 6, 337 25610394 10.3389/fnagi.2014.00337 PMC4285138

[122] Moyer J.R. Jr., Power J.M., Thompson L.T. and Disterhoft J.F. (2000) Increased excitability of aged rabbit CA1 neurons after trace eyeblink conditioning. J. Neurosci. 20, 5476–5482 10.1523/JNEUROSCI.20-14-05476.2000 10884331 PMC6772307

[123] Simkin D., Hattori S., Ybarra N., Musial T.F., Buss E.W., Richter H. et al. (2015) Aging-related hyperexcitability in CA3 pyramidal neurons is mediated by enhanced A-type K+ channel function and expression. J. Neurosci. 35, 13206–13218 10.1523/JNEUROSCI.0193-15.2015 26400949 PMC4579378

[124] Buss E.W., Corbett N.J., Roberts J.G., Ybarra N., Musial T.F., Simkin D. et al. (2021) Cognitive aging is associated with redistribution of synaptic weights in the hippocampus. Proc. Natl. Acad. Sci. U. S. A. 118, e1921481118 10.1073/pnas.1921481118 33593893 PMC7923642

[125] Xu B., Sun A., He Y., Qian F., Xi S., Long D. et al. (2018) Loss of thin spines and small synapses contributes to defective hippocampal function in aged mice. Neurobiol. Aging 71, 91–104 10.1016/j.neurobiolaging.2018.07.010 30118927

[126] Smith T.D., Adams M.M., Gallagher M., Morrison J.H. and Rapp P.R. (2000) Circuit-specific alterations in hippocampal synaptophysin immunoreactivity predict spatial learning impairment in aged rats. J. Neurosci. 20, 6587–6593 10.1523/JNEUROSCI.20-17-06587.2000 10964964 PMC6772954

[127] Magnusson K.R., Nelson S.E. and Young A.B. (2002) Age-related changes in the protein expression of subunits of the NMDA receptor. Brain Res. Mol. Brain Res. 99, 40–45 10.1016/S0169-328X(01)00344-8 11869807

[128] Kang H.J., Kawasawa Y.I., Cheng F., Zhu Y., Xu X., Li M. et al. (2011) Spatio-temporal transcriptome of the human brain. Nature 478, 483–489 10.1038/nature10523 22031440 PMC3566780

[129] Lu T., Pan Y., Kao S.Y., Li C., Kohane I., Chan J. et al. (2004) Gene regulation and DNA damage in the ageing human brain. Nature 429, 883–891 10.1038/nature02661 15190254

[130] Song L., Pan S., Zhang Z., Jia L., Chen W.H. and Zhao X.M. (2021) STAB: a spatio-temporal cell atlas of the human brain. Nucleic Acids Res. 49, D1029–D1037 10.1093/nar/gkaa762 32976581 PMC7778989

[131] Tabula Muris C. (2020) A single-cell transcriptomic atlas characterizes ageing tissues in the mouse. Nature 583, 590–595 10.1038/s41586-020-2496-1 32669714 PMC8240505

[132] Ximerakis M., Lipnick S.L., Innes B.T., Simmons S.K., Adiconis X., Dionne D. et al. (2019) Single-cell transcriptomic profiling of the aging mouse brain. Nat. Neurosci. 22, 1696–1708 10.1038/s41593-019-0491-3 31551601

[133] Nowakowski T.J., Bhaduri A., Pollen A.A., Alvarado B., Mostajo-Radji M.A., Di Lullo E. et al. (2017) Spatiotemporal gene expression trajectories reveal developmental hierarchies of the human cortex. Science 358, 1318–1323 10.1126/science.aap8809 29217575 PMC5991609

[134] Andrews B., Murphy A.E., Stofella M., Maslen S., Almeida-Souza L., Skehel J.M. et al. (2021) Multidimensional dynamics of the proteome in the neurodegenerative and aging mammalian brain. Mol. Cell. Proteom. 21, 100192 10.1016/j.mcpro.2021.100192 34979241 PMC8816717

[135] Carlyle B.C., Kandigian S.E., Kreuzer J., Das S., Trombetta B.A., Kuo Y. et al. (2021) Synaptic proteins associated with cognitive performance and neuropathology in older humans revealed by multiplexed fractionated proteomics. Neurobiol. Aging 105, 99–114 10.1016/j.neurobiolaging.2021.04.012 34052751 PMC8338777

[136] Graham L.C., Naldrett M.J., Kohama S.G., Smith C., Lamont D.J., McColl B.W. et al. (2019) Regional molecular mapping of primate synapses during normal healthy aging. Cell Rep. 27, 1018–+ 10.1016/j.celrep.2019.03.096 31018120 PMC6486486

[137] Fitzner D., Bader J.M., Penkert H., Bergner C.G., Su M., Weil M.T. et al. (2020) Cell-type- and brain-region-resolved mouse brain lipidome. Cell Rep. 32, 108132 10.1016/j.celrep.2020.108132 32937123

[138] Zocher S., Overall R.W., Lesche M., Dahl A. and Kempermann G. (2021) Environmental enrichment preserves a young DNA methylation landscape in the aged mouse hippocampus. Nat. Commun. 12, 3892 10.1038/s41467-021-23993-1 34162876 PMC8222384

[139] Ding J., Ji J., Rabow Z., Shen T., Folz J., Brydges C.R. et al. (2021) A metabolome atlas of the aging mouse brain. Nat. Commun. 12, 6021 10.1038/s41467-021-26310-y 34654818 PMC8519999

[140] Ben-Zvi A., Miller E.A. and Morimoto R.I. (2009) Collapse of proteostasis represents an early molecular event in Caenorhabditis elegans aging. Proc. Natl. Acad. Sci. U. S. A. 106, 14914–14919 10.1073/pnas.0902882106 19706382 PMC2736453

[141] Walther D.M., Kasturi P., Zheng M., Pinkert S., Vecchi G., Ciryam P. et al. (2015) Widespread Proteome remodeling and aggregation in aging C. Elegans. Cell 161, 919–932 10.1016/j.cell.2015.03.032 25957690 PMC4643853

[142] Yang F.Y., Chu X.L., Yin M.M., Liu X.L., Yuan H.R., Niu Y.M. et al. (2014) mTOR and autophagy in normal brain aging and caloric restriction ameliorating age-related cognition deficits. Behav. Brain Res. 264, 82–90 10.1016/j.bbr.2014.02.005 24525424

[143] Feleciano D.R., Juenemann K., Iburg M., Bras I.C., Holmberg C.I. and Kirstein J. (2019) Crosstalk between chaperone-mediated protein disaggregation and proteolytic pathways in aging and disease. Front. Aging Neurosci. 11, 10.3389/fnagi.2019.00009 30760997 PMC6361847

[144] Lipinski M.M., Zheng B., Lu T., Yan Z.Y., Py B.F., Ng A. et al. (2010) Genome-wide analysis reveals mechanisms modulating autophagy in normal brain aging and in Alzheimer's disease. Proc. Natl. Acad. Sci. U. S. A. 107, 14164–14169 10.1073/pnas.1009485107 20660724 PMC2922576

[145] Fernández Á F., Sebti S., Wei Y., Zou Z., Shi M., McMillan K.L. et al. (2018) Disruption of the beclin 1-BCL2 autophagy regulatory complex promotes longevity in mice. Nature 558, 136–140 10.1038/s41586-018-0162-7 29849149 PMC5992097

[146] Aman Y., Schmauck-Medina T., Hansen M., Morimoto R.I., Simon A.K., Bjedov I. et al. (2021) Autophagy in healthy aging and disease. Nat. Aging 1, 634–650 10.1038/s43587-021-00098-4 34901876 PMC8659158

[147] Glatigny M., Moriceau S., Rivagorda M., Ramos-Brossier M., Nascimbeni A.C., Lante F. et al. (2019) Autophagy is required for memory formation and reverses age-related memory decline. Curr. Biol. 29, 435.e8–448.e8 10.1016/j.cub.2018.12.021 30661803

[148] Pandey K., Yu X.W., Steinmetz A. and Alberini C.M. (2021) Autophagy coupled to translation is required for long-term memory. Autophagy 17, 1614–1635 10.1080/15548627.2020.1775393 32501746 PMC8354608

[149] Gupta V.K., Scheunemann L., Eisenberg T., Mertel S., Bhukel A., Koemans T.S. et al. (2013) Restoring polyamines protects from age-induced memory impairment in an autophagy-dependent manner. Nat. Neurosci. 16, 1453–+ 10.1038/nn.3512 23995066

[150] Stavoe A.K., Gopal P.P., Gubas A., Tooze S.A. and Holzbaur E.L. (2019) Expression of WIPI2B counteracts age-related decline in autophagosome biogenesis in neurons. Elife 8, 10.7554/eLife.44219 31309927 PMC6634969

[151] Kiffin R., Kaushik S., Zeng M., Bandyopadhyay U., Zhang C., Massey A.C. et al. (2007) Altered dynamics of the lysosomal receptor for chaperone-mediated autophagy with age. J. Cell Sci. 120, 782–791 10.1242/jcs.001073 17284523

[152] Xilouri M., Brekk O.R., Polissidis A., Chrysanthou-Piterou M., Kloukina I. and Stefanis L. (2016) Impairment of chaperone-mediated autophagy induces dopaminergic neurodegeneration in rats. Autophagy 12, 2230–2247 10.1080/15548627.2016.1214777 27541985 PMC5103347

[153] Xilouri M., Brekk O.R., Landeck N., Pitychoutis P.M., Papasilekas T., Papadopoulou-Daifoti Z. et al. (2013) Boosting chaperone-mediated autophagy in vivo mitigates alpha-synuclein-induced neurodegeneration. Brain 136, 2130–2146 10.1093/brain/awt131 23757764

[154] Hu Y.B., Dammer E.B., Ren R.J. and Wang G. (2015) The endosomal-lysosomal system: from acidification and cargo sorting to neurodegeneration. Transl. Neurodegener 4, 18 10.1186/s40035-015-0041-1 26448863 PMC4596472

[155] Lin H., Tang M., Ji C., Girardi P., Cvetojevic G., Chen D. et al. (2021) BAG3 regulation of RAB35 mediates the endosomal sorting complexes required for transport/endolysosome pathway and tau clearance. Biol. Psychiatry 92, 10–24 10.1016/j.biopsych.2021.10.024 PMC9085972 35000752

[156] Zhou J., Chow H.M., Liu Y., Wu D., Shi M., Li J. et al. (2020) Cyclin-dependent kinase 5-dependent BAG3 degradation modulates synaptic protein turnover. Biol. Psychiatry 87, 756–769 10.1016/j.biopsych.2019.11.013 31955914

[157] Tang M., Ji C., Pallo S., Rahman I. and Johnson G.V.W. (2018) Nrf2 mediates the expression of BAG3 and autophagy cargo adaptor proteins and tau clearance in an age-dependent manner. Neurobiol. Aging 63, 128–139 10.1016/j.neurobiolaging.2017.12.001 29304346 PMC5801049

[158] Abubakar Y.S., Zheng W., Olsson S. and Zhou J. (2017) Updated insight into the physiological and pathological roles of the retromer complex. Int. J. Mol. Sci. 18, 10.3390/ijms18081601 PMC5577995 28757549

[159] Choy R.W.Y., Park M., Temkin P., Herring B.E., Marley A., Nicoll R.A. et al. (2014) Retromer mediates a discrete route of local membrane delivery to dendrites. Neuron 82, 55–62 10.1016/j.neuron.2014.02.018 24698268 PMC4029335

[160] Mikhaylova M., Bera S., Kobler O., Frischknecht R. and Kreutz M.R. (2016) A dendritic golgi satellite between ERGIC and retromer. Cell Reports 14, 189–199 10.1016/j.celrep.2015.12.024 26748700

[161] Wu S.J., Fagan R.R., Uttamapinant C., Lifshitz L.M., Fogarty K.E., Ting A.Y. et al. (2017) The dopamine transporter recycles via a retromer-dependent postendocytic mechanism: tracking studies using a novel fluorophore-coupling approach. J. Neurosci. 37, 9438–9452 10.1523/JNEUROSCI.3885-16.2017 28847807 PMC5618262

[162] Chu J. and Pratico D. (2017) The retromer complex system in a transgenic mouse model of AD: influence of age. Neurobiol. Aging 52, 32–38 10.1016/j.neurobiolaging.2016.12.025 28110103

[163] Vagnozzi A.N. and Pratico D. (2019) Endosomal sorting and trafficking, the retromer complex and neurodegeneration. Mol. Psychiatr. 24, 857–868 10.1038/s41380-018-0221-3 PMC6378136 30120416

[164] Inoshita T., Arano T., Hosaka Y., Meng H.R., Umezaki Y., Kosugi S. et al. (2017) Vps35 in cooperation with LRRK2 regulates synaptic vesicle endocytosis through the endosomal pathway in Drosophila. Hum. Mol. Genet. 26, 2933–2948 10.1093/hmg/ddx179 28482024

[165] Ye H., Ojelade S.A., Li-Kroeger D., Zuo Z.Y., Wang L.P., Li Y.R. et al. (2020) Retromer subunit, VPS29, regulates synaptic transmission and is required for endolysosomal function in the aging brain. Elife 9, e51977 10.7554/eLife.51977 32286230 PMC7182434

[166] Vazquez-Sanchez S., Bobeldijk S., Dekker M.P., van Keimpema L. and van Weering J.R.T. (2018) VPS35 depletion does not impair presynaptic structure and function. Sci. Rep. 8, 2996 10.1038/s41598-018-20448-4 29445238 PMC5812998

[167] Fletcher B.R., Hill G.S., Long J.M., Gallagher M., Shapiro M.L. and Rapp P.R. (2014) A fine balance: Regulation of hippocampal Arc/Arg3.1 transcription, translation and degradation in a rat model of normal cognitive aging. Neurobiol. Learn. Mem. 115, 58–67 10.1016/j.nlm.2014.08.007 25151943 PMC4250373

[168] Koyuncu S., Loureiro R., Lee H.J., Wagle P., Krueger M. and Vilchez D. (2021) Rewiring of the ubiquitinated proteome determines ageing in C. elegans. Nature 596, 285–290 10.1038/s41586-021-03781-z 34321666 PMC8357631

[169] Reddy Addi U., Jakhotia S., Reddy S.S. and Reddy G.B. (2022) Age-related neuronal damage by advanced glycation end products through altered proteostasis. Chem. Biol. Interact. 355, 109840 10.1016/j.cbi.2022.109840 35104490

[170] Yin P., Tu Z., Yin A., Zhao T., Yan S., Guo X. et al. (2015) Aged monkey brains reveal the role of ubiquitin-conjugating enzyme UBE2N in the synaptosomal accumulation of mutant huntingtin. Hum. Mol. Genet. 24, 1350–1362 10.1093/hmg/ddu544 25343992 PMC4321442

[171] Giannini C., Kloss A., Gohlke S., Mishto M., Nicholson T.P., Sheppard P.W. et al. (2013) Poly-Ub-substrate-degradative activity of 26S proteasome is not impaired in the aging rat brain. PloS ONE 8, e64042 10.1371/journal.pone.0064042 23667697 PMC3646778

[172] Sacramento E.K., Kirkpatrick J.M., Mazzetto M., Baumgart M., Bartolome A., Di Sanzo S. et al. (2020) Reduced proteasome activity in the aging brain results in ribosome stoichiometry loss and aggregation. Mol. Syst. Biol. 16, e9596 32558274 10.15252/msb.20209596 PMC7301280

[173] Thibaudeau T.A., Anderson R.T. and Smith D.M. (2018) A common mechanism of proteasome impairment by neurodegenerative disease-associated oligomers. Nat. Commun. 9, 1097 10.1038/s41467-018-03509-0 29545515 PMC5854577

[174] Munkácsy E., Chocron E.S., Quintanilla L., Gendron C.M., Pletcher S.D. and Pickering A.M. (2019) Neuronal-specific proteasome augmentation via Prosβ5 overexpression extends lifespan and reduces age-related cognitive decline. Aging Cell. 18, e13005 10.1111/acel.13005 31334599 PMC6718538

[175] Grant S.G.N. and Fransen E. (2020) The synapse diversity dilemma: molecular heterogeneity confounds studies of synapse function. Front. Synaptic Neurosci. 12, 590403 10.3389/fnsyn.2020.590403 33132891 PMC7561708

[176] Mattson M.P. and Magnus T. (2006) Ageing and neuronal vulnerability. Nat. Rev. Neurosci. 7, 278–294 10.1038/nrn1886 16552414 PMC3710114

[177] Keller J.N., Hanni K.B. and Markesbery W.R. (2000) Possible involvement of proteasome inhibition in aging: implications for oxidative stress. Mech. Ageing Dev. 113, 61–70 10.1016/S0047-6374(99)00101-3 10708250

[178] Pluvinage J.V. and Wyss-Coray T. (2020) Systemic factors as mediators of brain homeostasis, ageing and neurodegeneration. Nat. Rev. Neurosci. 21, 93–102 10.1038/s41583-019-0255-9 31913356

[179] Escobar K.A., Cole N.H., Mermier C.M. and VanDusseldorp T.A. (2019) Autophagy and aging: Maintaining the proteome through exercise and caloric restriction. Aging Cell. 18, 10.1111/acel.12876 30430746 PMC6351830

